# The Effect of Single Nucleotide Polymorphisms from Genome Wide Association Studies in Multiple Sclerosis on Gene Expression

**DOI:** 10.1371/journal.pone.0010142

**Published:** 2010-04-13

**Authors:** Adam E. Handel, Lahiru Handunnetthi, Antonio J. Berlanga, Corey T. Watson, Julia M. Morahan, Sreeram V. Ramagopalan

**Affiliations:** 1 Wellcome Trust Centre for Human Genetics, University of Oxford, Oxford, United Kingdom; 2 Department of Clinical Neurology, University of Oxford, Oxford, United Kingdom; 3 Department of Biological Sciences, Simon Fraser University, Burnaby, British Columbia, Canada; 4 Blizard Institute of Cell and Molecular Science, Barts and The London School of Medicine and Dentistry, Queen Mary University of London, London, United Kingdom; Health Canada, Canada

## Abstract

**Background:**

Multiple sclerosis (MS) is a complex neurological disorder. Its aetiology involves both environmental and genetic factors. Recent genome-wide association studies have identified a number of single nucleotide polymorphisms (SNPs) associated with susceptibility to (MS). We investigated whether these genetic variations were associated with alteration in gene expression.

**Methods/Principal Findings:**

We used a database of mRNA expression and genetic variation derived from immortalised peripheral lymphocytes to investigate polymorphisms associated with MS for correlation with gene expression. Several SNPs were found to be associated with changes in expression: in particular two with *HLA-DQA1*, *HLA-DQA2*, *HLA-DQB1*, *HLA-DRB1*, *HLA-DRB4* and *HLA-DRB5*, one with *ZFP57*, one with *CD58*, two with *IL7* and *FAM164A*, and one with *FAM119B*, *TSFM* and *KUB3*. We found minimal cross-over with a recent whole genome expression study in MS patients.

**Discussion:**

We have shown that many susceptibility loci in MS are associated with changes in gene expression using an unbiased expression database. Several of these findings suggest novel gene candidates underlying the effects of MS-associated genetic variation.

## Introduction

Multiple sclerosis (MS) is an inflammatory disease of the central nervous system characterised by demyelination and axonal loss.[1] Studies conducted in mono- and dizygotic twin pairs and siblings have shown that genetics plays a role in MS susceptibility.[2] Linkage was effective in identifying the locus exerting the single strongest genetic effect in MS, namely, the human leukocyte antigen (HLA) class II region.[3] The risk associated with this region has since been shown to be determined by epistatic interactions between different HLA alleles,[4] and is thought to be responsible for approximately 50% of the genetic risk of MS.[5] Beyond this powerful determinant of MS genetic susceptibility, research has taken considerably longer to bear useful fruit. Finally, after the genotyping of hundreds of thousands of single nucleotide polymorphisms (SNPs) in many thousands of MS patients and controls, we are beginning to establish a network of loci outside of the HLA region involved in determining MS susceptibility.[6], [7], [8], [9], [10], [11], [12], [13], [14], [15], [16], [17] It is worth considering that even the most strongly associated of these with MS is still a significantly weaker determinant of MS susceptibility than HLA alleles. For some of these loci, functional studies have been undertaken.[11], [18], [19] However, such studies are rarely carried out in an unbiased manner since these generally correlate genetic variations with the expression of a candidate gene. A recent study of mRNA levels in MS patients and healthy controls showed a great multitude of differentially expressed genes however it is uncertain to what extent this reflects the aetiology of disease as opposed to the disease process or adaptive biological pathways.[20]

A recent investigation has performed whole genome expression analysis in lymphoblastoid cell lines (LCLs) from healthy volunteers who were also genotyped for a large number of SNPs.[21] We used the data from this study to examine the effects of current susceptibility loci in MS on gene expression.

## Methods

### Gene expression analysis

This was carried out as described in Dixon *et al.* [21] Briefly, peripheral lymphocytes were transformed using Epstein-Barr virus before being cultured, pelleted and frozen for storage. cDNA templates were created using the One-Cycle cDNA Synthesis Kit (Affymetrix). *In vitro* transcription of cDNA was performed using the IVT Labeling Kit (Affymetrix) and, after hybridisation on U133 Plus 2.0 GeneChips (Affymetrix), this was scanned using a high-resolution scanner (Affymetrix). Whole-genome genotyping was carried out according to manufacturers' instructions using the Sentrix Human-1 Genotyping BeadChip and the HumanHap300 Genotyping BeadChip. The analysis of expression was carried out on the publically available database of mRNA by SNP Browser 1.0 as described.[21]

### mRNA by SNP analysis

We investigated the mRNAs significantly altered in expression by the SNPs reported in the literature to be at or close to genome-wide significance.[6], [7], [8], [9], [10], [11], [12], [13], [14], [15], [16], [17] If the susceptibility SNP was not available on the database, we used the SNP with the strongest linkage disequilibrium (LD) with the susceptibility SNP as provided by SNP Browser 1.0 based on r^2^. For SNPs where no proxy was provided, we investigated all genotyped SNPs within 500 kb for LD with r^2^≥0.4 for a suitable proxy SNP. We also assessed the degree of LD with potentially interesting SNPs within 500 kb of the original susceptibility SNP. Finally we assessed the SNPs associated with expression of putative candidate genes to ensure that we did not miss any important associations with expression.

## Results

### SNP selection

We chose to look at a set of 38 SNPs which were the top loci to reach genome-wide significance selected from currently reported genome wide association studies (GWAS) of which 14 had been independently replicated in 2 studies. 17 of these were not present in the genome-wide association mRNA expression library and so when possible proxy SNPs in strong-to-moderate LD were used instead. The SNPs and proxy SNPs used are detailed in **Table 1**.

**Table 1:** SNPs and proxy SNPs analysed.

SNP	putative gene association	proxy SNP	r^2^
rs1054283	IL7		
rs10876994	METTL1, CYP27B1, CDK4	rs10083154	0.70067
rs1132200	TMEM39A		
rs11554159	IFI30	rs874628	0.949
rs11808092	EVI5-RPL5		
rs11865121	CLEC16A	rs2041670	1
rs12122721	KIF21B		
rs12368653	METTL1, CYP27B1, CDK4		
rs12708716	CLEC16A	rs725613	1
rs12722489	IL2RA	rs12722561	0.9349
rs1335532	CD58	rs6677309	1
rs1569723	CD40		
rs17445836	IRF8		
rs17824933	CD6	rs2237997	0.43718
rs1800693	TNFRSF1A		
rs2051322	CD226		
rs2104286	IL2RA	rs12722561	0.548
rs2300747	CD58	rs6677309	1
rs2523393	HLA class I	rs2394160	0.96552
rs2587156	IL7		
rs3129860	HLA class II	rs9271366	0.95557
rs3129934	C6orf10, BTNL2, NOTCH4	rs9267992	0.95604
rs3135388	HLA class II	rs9271366	0.95699
rs34536443	TYK2	No proxy available	
rs4149584	TNFRSF1A	No proxy available	
rs441349	SOCS1	rs1646042	1
rs6074022	CD40		
rs6131010	CD40	rs6074022	0.70337
rs6604026	EVI5-RPL5		
rs6860438	C7		
rs6897932	IL7R		
rs703842	METTL1, CYP27B1, CDK4		
rs7404554	CLEC16A		
rs744166	STAT3		
rs763361	CD226		
rs8118449	TYK2	No proxy available	
rs9271366	HLA class II		
rs9523762	GPC5		

### mRNA expression

13 of the MS susceptibility SNPs or proxy SNPs were associated with changes in mRNA expression (**Table S1**). Two SNPs in strong LD with multiple MS-associated SNPs in the HLA region were related to expression of various HLA alleles, including *HLA-DQA1*, *HLA-DQA2*, *HLA-DQB1*, *HLA-DRB1*, *HLA-DRB4* and *HLA-DRB5*. One SNP in the HLA class I region was associated with altered expression of *ZFP57*. Both SNPs in CD58 were associated with expression of *CD58*. A SNP in the IL7 region was associated with expression of mRNA encoding *IL7* and *FAM164A*. Three SNPs in the region of METTL1-CYP27B1-CDK4 altered the expression of several genes: *FAM119B*, *TSFM* and *KUB3*. The common gene of altered expression for all three SNPs was *TSFM*.

### Overlap with previous mRNA expression studies

We used the supplemental data supplied by Gandhi and colleagues to examine cross-over between the results obtained in that study and the genes we identified as being altered in expression by susceptibility SNPs.[20] Only three genes were in common between the two sets: *HLA-DQB1*, *HLA-DRB1* and *STAT3*. *HLA-DRB1* was upregulated in MS, relapsing-remitting MS (RRMS) and secondary progressive MS compared with healthy controls. *HLA-DQB1* expression was reduced in MS and RRMS compared with healthy controls. *STAT3* was reduced in primary progressive MS compared with healthy controls.

## Discussion

Our findings show that some, but by no means all, susceptibility SNPs in MS are associated with changes in gene expression. Some of these (*CD58*, had already been noted by previous investigators.[11] We were unable to find supporting evidence in this dataset for the previously reported allelic effect of the susceptibility SNP in *IL7R* on expression of the gene.[18] Similarly, SNPs in the *IL2RA* gene did not correlate with expression of *IL2RA* mRNA, despite previously finding altered levels of this in MS patients relative to controls.[19]

We also found several novel effects of susceptibility SNPs. Two SNPs in tight LD with susceptibility SNPs in the *HLA* region correlated with expression of several *HLA* class II mRNAs. However, measuring gene expression in the HLA is a complex task. There is haplotype specificity for some genes (*HLA-DRB4* and *HLA-DRB5*) and thus we are not sure whether differential expression of HLA genes measured by microarray reflects different probe affinity for different HLA class II alleles and thus further work is needed to fully understand this association. Our identification that a SNP in the *HLA* class I region was associated with altered expression of *ZFP57* is an interesting observation as this gene has been linked with DNA methylation changes across the genome resulting in transient neonatal diabetes.[22] There is some epidemiological evidence that MS may be partly determined by epigenetic alterations and this would be an ideal candidate functionally linking MS to the epigenome.[23] A SNP in *IL7* recently confirmed as associated with MS was shown to correlate with the expression of several genes: *IL7* and *FAM164A*. Naturally the most compelling candidate of these is *IL7* due to its probable role in autoimmunity. However, the advantage of an unbiased screen is that it raises the possibility of candidate genes that would otherwise not be considered. This is especially so since the SNP is far more strongly associated with *FAM164A* expression than with *IL7*. *FAM164A* is a hypothetical protein encoded in the reverse direction to *IL7* and its functional importance is largely unknown.[24] The susceptibility region on chromosome 12 was previously linked with the expression of *FAM119B*.[20] We feel that the relationship of all three major susceptibility SNPs with the expression of *TSFM* suggests this as a strong candidate. This is a plausible candidate in terms of function too as it is involved in the translation of mitochondrial proteins, providing a potential link with other susceptibility genes linked to mitochondrial function, such as *KIF21B*.[8], [25] Further functional work will be needed to better assess these candidates.

The limited cross-over between known and suspected susceptibility genes in the whole genome expression analysis of Gandhi and colleagues is likely due to a number of differences including the use of whole blood mRNA and individuals with established disease in the Gandhi study.[20] It is possible that future whole genome analyses of expression conducted using RNA-seq in cell-sorted samples of patients with very early disease may reveal alterations in the level of susceptibility gene mRNA.

The advantage of an unbiased approach to linking the expression of genes with genetic variation associated with disease susceptibility is that there is no *a priori* hypothesis to blind investigators to the presence of other genes. There are several limitations to the approach we used. The mRNA screen was conducted in transformed LCLs and so it would not be informative about tissue-specific gene expression.[21] Also, SNP coverage across the genome was not complete and so the functional effects of some SNPs for which no proxy was available will be concealed. Furthermore, despite using expression data from 400 LCLs, we may have been underpowered to detect relevant effects. However, our finding of several novel associations between MS SNPs and gene expression is worthy of further investigation and also raises the hypothesis that some disease associated SNPs may not exert their effects on MS susceptibility through simple effects on gene expression.

## Supporting Information

Table S1 Changes in mRNA expression associated with susceptibility SNPs.
(0.15 MB DOC)

## References

[1] Noseworthy JH, Lucchinetti C, Rodriguez M, Weinshenker BG (2000). Multiple sclerosis.. N Engl J Med.

[2] Willer CJ, Dyment DA, Risch NJ, Sadovnick AD, Ebers GC (2003). Twin concordance and sibling recurrence rates in multiple sclerosis.. Proceedings of the National Academy of Sciences of the United States of America.

[3] Ebers GC, Kukay K, Bulman DE, Sadovnick AD, Rice G (1996). A full genome search in multiple sclerosis.. Nature Genetics.

[4] Lincoln MR, Ramagopalan SV, Chao MJ, Herrera BM, Deluca GC (2009). Epistasis among HLA-DRB1, HLA-DQA1, and HLA-DQB1 loci determines multiple sclerosis susceptibility.. Proc Natl Acad Sci U S A.

[5] Peltonen L (2007). Old suspects found guilty–the first genome profile of multiple sclerosis.. The New England Journal of Medicine.

[6] Burton PR, Clayton DG, Cardon LR, Craddock N, Deloukas P (2007). Association scan of 14,500 nonsynonymous SNPs in four diseases identifies autoimmunity variants.. Nature Genetics.

[7] Hafler DA, Compston A, Sawcer S, Lander ES, Daly MJ (2007). Risk alleles for multiple sclerosis identified by a genomewide study.. The New England Journal of Medicine.

[8] IMSGC (2010). Comprehensive follow-up of the first genome-wide association study of multiple sclerosis identifies KIF21B and TMEM39A as susceptibility loci.. Hum Mol Genet.

[9] Ban M, Goris A, Lorentzen AR, Baker A, Mihalova T (2009). Replication analysis identifies TYK2 as a multiple sclerosis susceptibility factor.. Eur J Hum Genet.

[10] De Jager PL, Jia X, Wang J, de Bakker PI, Ottoboni L (2009). Meta-analysis of genome scans and replication identify CD6, IRF8 and TNFRSF1A as new multiple sclerosis susceptibility loci.. Nat Genet.

[11] De Jager PL, Baecher-Allan C, Maier LM, Arthur AT, Ottoboni L (2009). The role of the CD58 locus in multiple sclerosis.. Proceedings of the National Academy of Sciences of the United States of America.

[12] Hafler JP, Maier LM, Cooper JD, Plagnol V, Hinks A (2009). CD226 Gly307Ser association with multiple autoimmune diseases.. Genes Immun.

[13] Baranzini SE, Wang J, Gibson RA, Galwey N, Naegelin Y (2009). Genome-wide association analysis of susceptibility and clinical phenotype in multiple sclerosis.. Hum Mol Genet.

[14] Rubio JP, Stankovich J, Field J, Tubridy N, Marriott M (2008). Replication of KIAA0350, IL2RA, RPL5 and CD58 as multiple sclerosis susceptibility genes in Australians.. Genes Immun.

[15] (ANZgene) AaNZMSGC (2009). Genome-wide association study identifies new multiple sclerosis susceptibility loci on chromosomes 12 and 20.. Nat Genet.

[16] Kallio SP, Jakkula E, Purcell S, Suvela M, Koivisto K (2009). Use of a genetic isolate to identify rare disease variants: C7 on 5p associated with MS.. Hum Mol Genet.

[17] Jakkula E, Leppa V, Sulonen AM, Varilo T, Kallio S (2010). Genome-wide association study in a high-risk isolate for multiple sclerosis reveals associated variants in STAT3 gene.. Am J Hum Genet.

[18] Gregory SG, Schmidt S, Seth P, Oksenberg JR, Hart J (2007). Interleukin 7 receptor alpha chain (IL7R) shows allelic and functional association with multiple sclerosis.. Nature Genetics.

[19] Maier LM, Lowe CE, Cooper J, Downes K, Anderson DE (2009). IL2RA genetic heterogeneity in multiple sclerosis and type 1 diabetes susceptibility and soluble interleukin-2 receptor production.. PLoS Genet.

[20] Gandhi KS, McKay FC, Cox M, Riveros C, Armstrong N (2010). The multiple sclerosis whole blood mRNA transcriptome and genetic associations indicate dysregulation of specific T cell pathways in pathogenesis.. Hum Mol Genet In press.

[21] Dixon AL, Liang L, Moffatt MF, Chen W, Heath S (2007). A genome-wide association study of global gene expression.. Nat Genet.

[22] Mackay DJ, Callaway JL, Marks SM, White HE, Acerini CL (2008). Hypomethylation of multiple imprinted loci in individuals with transient neonatal diabetes is associated with mutations in ZFP57.. Nat Genet.

[23] Handel AE, Ebers GC, Ramagopalan SV (2009). Epigenetics: molecular mechanisms and implications for disease.. Trends Mol Med.

[24] Kono T, Bird S, Sonoda K, Savan R, Secombes CJ (2008). Characterization and expression analysis of an interleukin-7 homologue in the Japanese pufferfish, Takifugu rubripes.. FEBS J.

[25] Smeitink JA, Elpeleg O, Antonicka H, Diepstra H, Saada A (2006). Distinct clinical phenotypes associated with a mutation in the mitochondrial translation elongation factor EFTs.. Am J Hum Genet.

