# Supplementary material for: The Effect of Single Nucleotide Polymorphisms from Genome Wide Association Studies in Multiple Sclerosis on Gene Expression
Source: PLoS One. 2010 Apr 13;5(4):e10142. doi: 10.1371/journal.pone.0010142 (PMC2854120; doi:10.1371/journal.pone.0010142)
Supplement: Table S1 — Changes in mRNA expression associated with susceptibility SNPs. (0.15 MB DOC) [file pone.0010142.s001.doc]

| **SNP** | **SNPs in LD** | **Transcript** | **Allele** | **Expression effect** | **P-value** | **Gene expressed** |
| --- | --- | --- | --- | --- | --- | --- |
| rs10083154 | rs10876994 | 213861_s_at | G | -0.412 | 3.50E-07 | FAM119B |
| rs10083154 | rs10876994 | 212656_at | G | 0.41 | 6.30E-07 | TSFM |
| rs1054283 |  | 205308_at | G | 0.872 | 1.60E-27 | FAM164A |
| rs1054283 |  | 234357_at | G | -0.86 | 2.90E-26 | --- |
| rs1054283 |  | AVG_C8ORF70 | G | 0.756 | 5.20E-21 | FAM164A |
| rs1054283 |  | 236215_at | G | 0.597 | 9.40E-14 | FAM164A |
| rs1054283 |  | AVG_CGI-62 | G | 0.499 | 6.20E-10 | FAM164A |
| rs1054283 |  | 241808_at | G | 0.433 | 3.60E-08 | IL7 |
| rs1132200 |  | None |  |  |  |  |
| rs11808092 |  | None |  |  |  |  |
| rs12122721 |  | None |  |  |  |  |
| rs12368653 |  | 227678_at | G | -0.374 | 1.10E-06 | KUB3 |
| rs12368653 |  | 212656_at | G | -0.394 | 3.10E-07 | TSFM |
| rs12368653 |  | AVG_KUB3 | G | -0.392 | 3.50E-07 | KUB3 |
| rs12722561 | rs12722489, rs2104286 | None |  |  |  |  |
| rs1569723 |  | None |  |  |  |  |
| rs1646042 | rs441349 | None |  |  |  |  |
| rs17445836 |  | None |  |  |  |  |
| rs1800693 |  | None |  |  |  |  |
| rs2041670 | rs11865121 | None |  |  |  |  |
| rs2051322 |  | None |  |  |  |  |
| rs2237997 | rs17824933 | None |  |  |  |  |
| rs2394160 | rs2523393 | 231236_at | A | 0.495 | 2.40E-10 | ZFP57 |
| rs2587156 |  | None |  |  |  |  |
| rs6074022 |  | None |  |  |  |  |
| rs6604026 |  | None |  |  |  |  |
| rs6677309 | rs1335532, rs2300747 | AVG_CD58 | A | -0.481 | 1.30E-06 | CD58 |
| rs6677309 | rs1335532, rs2300747 | AVG_CD58 | A | -0.481 | 1.30E-06 | CD58 |
| rs6860438 |  | None |  |  |  |  |
| rs6897932 |  | None |  |  |  |  |
| rs703842 |  | 213861_s_at | T | -0.695 | 7.80E-17 | FAM119B |
| rs703842 |  | 212656_at | T | 0.568 | 2.50E-11 | TSFM |
| rs725613 | rs12708716 | None |  |  |  |  |
| rs7404554 |  | None |  |  |  |  |
| rs744166 |  | None |  |  |  |  |
| rs763361 |  | None |  |  |  |  |
| rs874628 | rs11554159 | None |  |  |  |  |
| rs9267992 | rs3129934 | AVG_HLA-DQA1 | A | -0.621 | 1.80E-08 | HLA-DQA1 |
| rs9267992 | rs3129934 | 204670_x_at | A | 0.681 | 9.10E-10 | HLA-DRB1 |
| rs9267992 | rs3129934 | 209823_x_at | A | 0.576 | 2.20E-07 | HLA-DQB1 |
| rs9267992 | rs3129934 | AVG_HLA-DRB1 | A | 0.587 | 1.40E-07 | HLA-DRB1 |
| rs9267992 | rs3129934 | 209480_at | A | -0.935 | 8.60E-18 | HLA-DQB1, LOC650557 |
| rs9267992 | rs3129934 | 236203_at | A | -0.759 | 9.20E-12 | HLA-DQA1 |
| rs9267992 | rs3129934 | 212999_x_at | A | -0.855 | 1.60E-14 | HLA-DQB1, LOC390569 |
| rs9267992 | rs3129934 | 213831_at | A | -0.812 | 1.10E-13 | HLA-DQA1, HLA-DQA2 |
| rs9267992 | rs3129934 | 209312_x_at | A | 0.643 | 8.00E-09 | HLA-DRB5, HLA-DRB1 |
| rs9267992 | rs3129934 | 238900_at | A | -0.881 | 1.90E-15 | --- |
| rs9267992 | rs3129934 | 211654_x_at | A | -0.885 | 2.30E-15 | HLA-DQB1 |
| rs9267992 | rs3129934 | 217362_x_at | A | 0.625 | 1.90E-08 | HLA-DRB6, HLA-DRB1 |
| rs9267992 | rs3129934 | 212998_x_at | A | -0.593 | 1.20E-07 | HLA-DRB1, HLA-DRB4 |
| rs9271366 | rs3135388, rs3129860 | AVG_HLA-DQA1 | A | -0.598 | 1.20E-08 | HLA-DQA1 |
| rs9271366 | rs3135388, rs3129860 | 204670_x_at | A | 0.743 | 2.00E-12 | HLA-DRB1 |
| rs9271366 | rs3135388, rs3129860 | 209823_x_at | A | 0.624 | 3.50E-09 | HLA-DQB1 |
| rs9271366 | rs3135388, rs3129860 | AVG_HLA-DRB1 | A | 0.648 | 9.10E-10 | HLA-DRB1 |
| rs9271366 | rs3135388, rs3129860 | 209480_at | A | -0.882 | 1.70E-17 | HLA-DQB1, LOC650557 |
| rs9271366 | rs3135388, rs3129860 | 236203_at | A | -0.72 | 1.00E-11 | HLA-DQA1 |
| rs9271366 | rs3135388, rs3129860 | 212999_x_at | A | -0.809 | 2.00E-14 | HLA-DQB1, LOC390569 |
| rs9271366 | rs3135388, rs3129860 | 213831_at | A | -0.787 | 4.00E-14 | HLA-DQA1, HLA-DQA2 |
| rs9271366 | rs3135388, rs3129860 | 209312_x_at | A | 0.721 | 9.60E-12 | HLA-DRB5, HLA-DRB1 |
| rs9271366 | rs3135388, rs3129860 | 238900_at | A | -0.963 | 5.00E-20 | --- |
| rs9271366 | rs3135388, rs3129860 | 211654_x_at | A | -0.755 | 1.10E-12 | HLA-DQB1 |
| rs9271366 | rs3135388, rs3129860 | 217362_x_at | A | 0.67 | 2.00E-10 | HLA-DRB6, HLA-DRB1 |
| rs9271366 | rs3135388, rs3129860 | 208306_x_at | A | 0.573 | 5.80E-08 | HLA-DRB1, HLA-DRB4 |
| rs9523762 |  | None |  |  |  |  |
